# Relation between innovative work behavior and ethical climate perceptions among nursing personnel

**DOI:** 10.1186/s12912-024-01703-8

**Published:** 2024-02-13

**Authors:** Nagwa Gouda Ahmed Abd-Elmoghith, Amal Sobhy Mahmoud, Aida Mahmoud Abdel-Azeem

**Affiliations:** 1 Faculty of Nursing, Kafrelsheikh University, Kafrelsheikh, Egypt; 2 Psychiatric Nursing and Mental Health, Faculty of Nursing, Port Said University, Port Said, Egypt; 3 Faculty of Nursing, Beni-Suef University, Beni-Suef, Egypt

**Keywords:** Ethical climate, Innovative work behavior, Nursing personnel, Perception

## Abstract

**Background:**

Globalization and innovative technologies forced organizations to adopt innovative approaches and innovations for gaining a sustainable competitive advantage. Innovative Work Behavior (IWB) is related to the employees, ability, and enthusiasm to create innovative ideas. It exhibits a dynamic framework that is easier to be impacted by the ethical climate.

**Methods:**

Descriptive correlational design was applied and the study was performed at different inpatient units in Kafrelsheikh Governorate General Hospital. Two hundred twenty-two staff nurses and 45 head nurses from the aforementioned setting were chosen as a purposive sample. Two instruments were utilized to obtain the data; Innovative Work Behavior questionnaire and Ethical Climate Questionnaire. The significance of the acquired data was evaluated at the 5% level. Number and percentage were used to describe qualitative data and Range frequency, mean, standard deviation, and Pearson coefficient were used to characterize quantitative data.

**Results:**

More than half of staff nurses had a positive perception of innovative work behavior and more than three quarters of them had a negative Ethical climate perception.

**Conclusion:**

The study proved a significant relation between Efficiency dimension and the overall innovative work behavior perception *p* = 0.044, and the economic affairs and innovation dimension and the overall ethical climate perception *p* = 0.033.

## Background

In today’s extremely sensitive and modern environment, innovation is vital for the health sector’s survival and long-term competitive advantage [1]. Employees’ creative ideas and innovative potential have long been acknowledged as the process that drives organizational innovation at the individual level [2]. Logically individuals participate with a crucial part in inventing things as they are the new ideas, holders and processors, so they are considered the foundation of all innovations that are subsequently developed further [3].

Doctors indicate information acquisition abilities, whereas nurses show their abilities to generate fresh ideas that are more crucial for creativeness [4]. Nurses are the cornerstone of health industry [5] because they are vital to caring for patients and offering the 24- hour services [6, 7]. Since nurses provide up to eighty percent of the primary care and are predicted to work harder with fewer resources to meet the new requirements of the health sector, they are in a good position to make innovative contributions [8].

The term (IWB) is described as a person acting in a way that actively develops, introduces, and applies new concepts, procedures, or products[3]. Also, It is a cognitive and motivational process that incorporates workers’ capacity to produce, and implement new ideas at work as well as offer innovative and effective solutions to difficult problems [9, 10]. Moreover, it can be used to describe how much time and effort people invest in creating, enhancing, and putting innovative ideas into practice within their jobs [11, 12]. Usually, these creative work tasks exceed the responsibilities specified in job descriptions [13].

Early studies carried out by Kanter [14] and also Scott & Bruce [15] documented three components for the dimensions of IWB, including invention of innovative ideas, the creation of grouping organization members for the creative idea, and application of it in a larger context. Then, it was suggested that IWB has four stages [16]. Exploration of opportunities was added as the IWB’s fourth component because of having its unique challenges, as it necessitates monitoring the developments of the work, changes which occur in the structures of the organizations, in addition to the novel ideas of the individuals. In this setting, developing new ideas comes from critically analyzing both individual and collective beliefs and also involves addressing work problems. The promotion seeks to encourage innovative environments [13].

Organizations can promote innovative behaviors using two methods: perceived encouragement for innovation and providing sufficient assets. The first method is related to the individuals’ perceptions of the extent to which their organizations provide encouragement for innovations. It comprises promoting innovation throughout the entire organization, supporting and encouraging risk-taking. While, the second method refers to resources of the organization that enhance innovation such as supplying necessary facilities and equipment, as well as allowing time for innovation [17]. Also, supervisors can play great role to enhance IWB inside the organization through building an innovative climate [3].

According to Victor and Cullen [18], the ethical climate is defined as” All activities, rules and norms inside the organization that are within ethical substance” [19]. In healthcare, ethical climate can be defined as “All values either explicit or Implicit that guide the delivery of healthcare and form the place of work within which the care provided” [20].

Five categories of ethical environment are distinguished: caring of others, law and code, rules, instrumental, and independent. Nine sub dimensions are then generated in relation to these types: Efficiency, morality of the workforce, Self-interest, Procedures and rules of the organization, Friendship, profits of the organization, Professional codes and laws, responsibility toward society, and interest of the team [21]. The social context in which nursing is practiced involves the interaction of interpersonal connections and environmental elements. Therefore, the workplace environment has a significant impact on nurses’ behavior and nursing practice [3].

Egypt faces a crucial problem related to life threats and chronic fatal diseases such as cancer. This may be due to the few supplies and equipment, the lack of diagnosing these diseases at advanced stages and the limitation in sedations, accessibility and availability for medical use. So, palliative care and end-of-life (EOL) activities are one of ethically related issues which necessitate innovative ways and behaviors from nurses and other health professionals in order to increase satisfaction of patients, their families and nurses overall [22]. On the other hand, there are barriers for providing EOL care for dying patients such as poor design of ICUs units and nurses, work overload. It necessitates a change in the policies, education and the research of the Egyptian healthcare system for improving the nurses, perception about such innovative way [23].

In Egypt, healthcare services are managed, financed and provided by governmental or private institutions. The ministry of health and population is the essential provider of healthcare services in Egypt. Additionally, other institutions, such as the ministry of higher education and scientific research, ministry of defense and ministry of interior, also offer health services for their staff members. Across the country, the health workforce density is 13.5 physicians and 22.3 nurses per 10000 populations. Nurses represent the largest sector of the healthcare workforce [23].

The study implemented in a governmental hospital after the outbreak of covid-19 pandemic, where there was a diminishing in the tightening measures. Also, most of Egyptians had the vaccine. Generally, there are lack of resources and supplies; in addition to shortage of staff especially at governmental places.

### Significant of study

The growth of any organization especially healthcare ones depends on the innovative behavior of their employees. Nurses in particular must demonstrate innovative behaviors for a number of reasons, including the requirement to adapt to the shifting illness load such as covid-19, the fast technological advancements, novel care models and new communication and data processing approaches [1]. The ethical climate acts as a guide for staff members to understand the acceptable and unacceptable actions, assisting them in making more informed judgments. Moreover, it can affect the willingness of nurses to continue their job duties [24]. Therefore, this study was conducted to explore the relation between innovative work behavior and ethical climate perceptions among nursing personnel.

### Study aim

This study aims to explore the relation between innovative work behavior and ethical climate perceptions among nursing personnel.

### Research questions

1. Do the nursing personnel have a positive perception toward innovative work behavior and ethical climate?
2. Is there a relation between innovative work behavior and ethical climate perceptions among nursing personnel?

## Method

### Research design

Descriptive correlational research design was used.

### Research setting

Data were collected from different inpatient departments at Kafrelsheikh Governorate General Hospital that follows the Ministry of Health, Kafrelsheikh city, Kafrelsheikh Governorate, Egypt. The hospital is divided into four floors. The ground floor contains administrative hospital offices, inpatient, psychiatric, and mental male and female units. The first floor includes the intensive care unit (medical & surgical ICU), emergency surgical unit, inpatient surgical male unit, orthopedic unit, and Urology unit. The second floor contains operating rooms (ORs); burn unit, neonate intensive care unit (NICU), obstetric unit, and surgical female unit. The third floor includes diabetic unit, medical unit, neurosurgery ICU, pediatric unit, and pediatric intensive care unit (PICU).

### Research subjects

A purposive sample was used.

### Sample size

The ideal sample size was estimated at a confidence interval of 95%, margin of errors 5.0%, a total population size of (521 staff nurses, 51 head nurses), by using the following formula the researchers calculated the participants of staff nurses and head nurses separately [25]:

$$n=\frac{N\times p\left(1-p\right)}{\left[\left[N-1\times \left({d}^{2}\div {z}^{2}\right)\right]+p\left(1-p\right)\right]}$$

N = population size (521) staff nurses & (51) head nurses.

p = probability (50%)

Z: confidence level at 95% (1.96)

d: error proportion (0.05)

n = sample size: 222 (staff nurses) + 45 (head nurses) = 267 nursing personnel

Accordingly 267 nursing personnel They were chosen randomly from the total number (521 staff nurses and 51 head nurses) as follows: Burn (10 staff nurses, 3 head nurses), CCU (12 staff nurses, 3 head nurses), Dialysis (12 staff nurses, 3 head nurses), ICU (10 staff nurses, 3 head nurses), PICU (20 staff nurses, 4 head nurses), Incubator (20 staff nurses, 4 head nurses), Isolation ( 25 staff nurses, 4 head nurses), Medical department (15 staff nurses, 3 head nurses), Obstetric (16 staff nurses, 3 head nurses), OR (37 staff nurses, 6 head nurses), Orthopedic (10 staff nurses, 2 head nurses), Surgical ( 20 staff nurses, 4 head nurses) and Urology (15 staff nurses, 3 Head nurses).

The selected sample had the following inclusion criteria; accept to participate in the study, had at least one year experience in their work place and were available during data collection period. All staff nurses and head nurses who didn’t have the previously mentioned criteria were excluded from the study.

### Study tools

Data were collected by using two tools.

#### Tool I: Innovative Work Behavior Questionnaire (IWBQ)

It was developed by Oukes [16] in an English language and translated by a researcher in an Arabic language. The questionnaire aimed to determine innovative work behavior perception among nurses’ personnel. It was consisted of 76 items which constructed and divided into seven dimensions: Innovative work behavior daily activities (8 items); employee production (15 items); innovative output (17 items); expected positive performance outcomes (3 items); innovation stimulating leadership behavior (18 items); personnel policy (10 items) and economic affairs & innovation (5 items). The nursing personnel responses on a Likert scale ranged from 1 to 5 (where 1 is for strongly disagree and 5 is for strongly agree).

#### Scoring of IWBQ

The replies to all items were added up to produce a final score, and domains scores are obtained by summing the responses of items from each respective domain, then converted into a percentage. If the total percent score ≥ 60% is considered positive perception while < 60% was consider negative perception.

#### Tool II: Ethical climate questionnaire

This questionnaire was developed by Victor & Cullen [18], in an English language and translated by a researcher in an Arabic language; it was consisting of 36 items. The questionnaire aimed to assess ethical climate in organizations, all of which may be predicted to differ along these two dimensions—ethical criteria and locus of analysis as depicted in (Table 2).

This tool designed to ask respondents about their perception of how members of an organization normally decide on different “events, policies, and processes” needing ethical standards, it contained 36 items classified into sub dimensions in (Table 1) each sub dimensions contains items as: Self-interest (EI) = Four items (1, 6,10, 33), efficiency (EC) = Four items (2,19, 25, 36), friendship, team interest(BL) = eight items (5, 12,16, 21, 27, 31, 32, 35), social responsibility (BC) = Four items (26, 28, 30, 34), personal morality (PI) = Four items (3, 9, 11, 22), rules, standard operating procedures (PL) = Four items (7,15, 18, 23), laws, professional codes (PC) = Four items (13,14, 20, 24) and organizational profit (EL) = Four items (4, 8, 17, 29). The reverse items were (15, and 16). The nursing personnel responses on a Likert scale from 1 to 5 (where 1 is for strongly disagree and 5 is for strongly agree).

**Table 1:** Sub dimensions of ethical climate [18]

Ethical criteria	Locus of analysis		
	Individual (I)	Local (L)	Cosmopolitan (C)
Egoism (E)	Self-interest (EI)	Organizational Profit (EL)	Efficiency (EC)
Benevolence (B)	Friendship &Team Interest (BL)		Social Responsibility (BC)
Principle (P)	Personal Morality (PI)	Rules, Standard Operating Procedures (PL)	Laws, Professional Codes (PC)

#### Scoring of ethical climate questionnaire

The scores were summed, then converted into a percentage ≥ 60% is considered positive perception while < 60% was considered negative perception.

In addition to that, a Personal and Job Characteristics Sheet: This sheet was developed by a researcher in an Arabic language. It included personal characteristics such as nurses’ age, gender, educational qualification, and marital status. It also comprised questions that covering data related to job characteristics as years of experience, hospital name and department.

### Translation process

The questionnaires were translated into Arabic by the researchers to ensure that they were suitable to the Egyptian culture and the different educational levels of nurses. They were evaluated by a jury of five experts of academic members. According to their recommendations, some items were modified for more clarity. Then a back translation into English was conducted by a language expert. The researchers and the jury members reviewed the back translation to ensure the accuracy and validity of the tools.

### Tools validity

This stage was completed over time (two months). Following the translation, the questionnaires were reviewed by the previously mentioned jury to ensure that they were clear, comprehensive, constructive and simple.

### Tools reliability

The tools’ internal consistency and testing reliability were evaluated using Cronbach’s Alpha. The ethical climate questionnaire scored (0.878) on the Cronbach’s Alpha scale, which indicates reliable tools, while the innovative work behavior tool scored (0.917).

### Pilot study

Before beginning the main study, a pilot study with 10% of the participants (27 nurses) was carried out to assess the tools’ applicability and viability and make any necessary modifications. No nurses who had taken part in the pilot trial were included in the study sample.

### Field work

The field work of this study was implemented through two stages as follows:

- *First stage*: Before embarking on the field work, the total number of head nurses and staff nurses who are working in Kafrelsheikh Governorate General Hospital was obtained from Directorate of Health Affairs of the Hospital to estimate the sample size. The data was collected through three days per week (Saturday, Monday, and Wednesday). It was concerned with the preparation of the data collection tools that where it conducted over a period of four months commencing first of February 2022 to end of May 2022. A cover letter that described the study’s purpose to the participants was included with the questionnaire.
- *Second stage*: It sought to investigate the relationship between innovative work practices and nursing staff perceptions of the ethical climate. Following an explanation of the purpose and study tools, each participant completed the necessary tool in a face-to-face interview, taking an average of 20 to 30 min.

### Statistical analysis

Data were entered into the computer and assessed using the IBM SPSS software package, version 20.0. (IBM Corp; Armonk, New York). The terms used to describe qualitative data were number and percentage. The Kolmogorov-Smirnov test was used to determine whether the distribution was normal. There was no missing in the dataset. Quantitative data were described using range frequency, mean, standard deviation, and Pearson coefficient. The significance of the findings was established at the 5% level.

## Results

Table 2 shows that slightly less than half of nurses (46.4%) had age less than 20 years old. Regarding to gender, most of nurses (93.6%) were female. As regard educational qualification, it was noticed that 43.4% of nursing personnel had technical institute of nursing, while less than half of nurses (41.6%) had from 5- < 10 years of experience. Regarding to marital status, nearly the half (49.4%) of them were single.

**Table 2:** Frequency distribution of studied nursing personnel according to personal and job characteristics (*n* = 267)

Personal and job characteristics	Nurses’ personnel	
	**No**	**%**
**Nurses’ Age (years)**		
< 20	124	46.4
20 – < 25	71	26.6
25 – < 30	54	20.2
30 – < 35	12	4.5
35 – < 40	6	2.2
Range	18.0 – 39.0	
Mean ± SD	22.20 ± 4.75	
**Gender**		
Male	17	6.4
Female	250	93.6
**Educational Qualification**		
Secondary Nursing diploma	91	34.1
Technical institute of Nursing	116	43.4
Bachelor of Nursing	20	7.5
Another	40	15.0
**Marital Status**		
Single	132	49.4
Married	110	41.2
Divorced	19	7.1
Widow	6	2.2
**Years of Experience**		
1– < 5	96	36.0
5- < 10	111	41.6
10- < 15	58	21.7
≤ 15	2	0.7
Range	1.0 – 17.0	
Mean ± SD	7.29 ± 3.98	
**Job**		
Nurse	222	83.1
Head nurse	45	16.9

Table 3 displays that, most of the studied sample indicated that they had positive perception about expected positive performance outcomes dimension (70.4%). Meanwhile, less than two thirds (65.5%) of the studied sample indicated that their perception about personnel policy dimension was negative. Meanwhile, the overall Mean score ± SD of innovative work behavior perception was (259.36 ± 41.18). Also, the highest mean score of innovative work behavior perception 60.25 ± 8.91was related to innovative output dimension. Meanwhile, the lowest mean score percent (11.10 ± 2.40) was related to expected positive performance outcomes dimension.

**Table 3:** Distribution of the studied nursing personal perceptions according to innovative work behavior dimensions (*n* = 267)

Innovative work behavior dimensions	Range	Innovative work behavior perception				Mean score ± SD
		**Negative**		**Positive**		
		**No**	**%**	**No**	**%**	
Innovative work behavior daily activities	8 – 40	86	32.2	181	67.8	29.22 ± 4.75
Employee production	15 – 75	97	36.3	170	63.7	53.81 ± 9.47
Innovative output	17 – 85	85	31.8	182	68.2	60.25 ± 8.91
Expected positive performance outcomes	3 – 15	79	29.6	188	70.4	11.10 ± 2.40
Innovation stimulating leadership behavior	18 – 90	140	52.4	127	47.6	57.56 ± 15.69
Personnel policy	10 – 50	175	65.5	92	34.5	31.91 ± 7.24
Economic affairs & innovation	5 – 25	161	60.3	106	39.7	15.52 ± 4.65
**Overall innovative work behavior**	76 – 380	128	47.9	139	52.1	**259.36 ± 41.18**

***SD* Standard deviation**

**Fig. 1:**
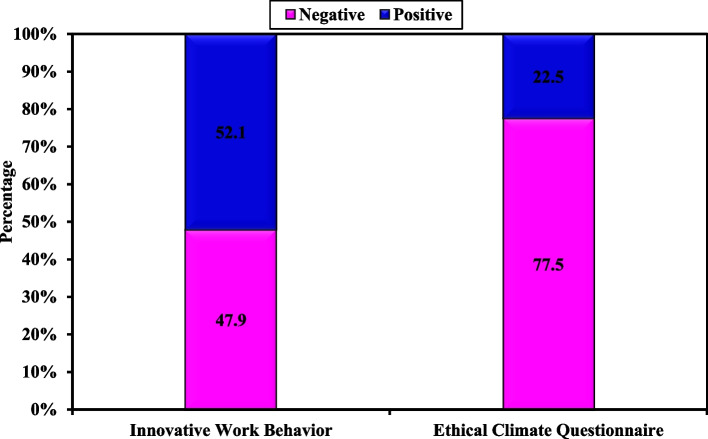
Descriptive analysis of the studied sample according to overall innovative work behavior tool (*n* = 267) and overall ethical climate questionnaire (*n* = 267)

Figure 1 indicates that more than half of staff nurses (57.3%) had a positive perception of overall innovative work behavior. Meanwhile, less than half (42.7%) of them had a negative perception of overall innovative work behavior perception. Regarding ethical climate perception, more than three quarters of nurses (77.53%) had a negative perception. Meanwhile, the lowest percent (22.47%) of them had a positive perception.

Table 4 reveals that, the majority of the studied sample (80.1%) indicated that their perception of the self- interest dimension was negative. Meanwhile, nearly the third of the studied sample (33.3%) indicated that their perception about efficiency dimension was positive. Furthermore, the overall Mean score ± SD of ethical climate dimensions was (109.48 ± 18.73). While the highest Mean score ± SD (21.09 ± 4.23) was related to Organization profit dimension. Meanwhile, the lowest Mean score ± SD (8.79 ± 2.45) was related to Laws, professional codes dimension.

**Table 4:** Distribution of the studied sample according to the ethical climate perception’s dimensions (*n* = 267)

Ethical climate dimensions	Range	Negative		Positive		Mean score ± SD
		**No**	**%**	**No**	**%**	
Self-interest (EI)	4 – 20	214	80.1	53	19.9	11.71 ± 3.32
Efficiency (EC)	4 – 20	178	66.7	89	33.3	12.78 ± 2.47
Friendship, team interest (BI)	8 – 40	198	74.2	69	25.8	18.41 ± 3.79
Social responsibility (BC)	4 – 20	209	78.3	58	21.7	12.67 ± 2.58
Personal morality (PI)	4 – 20	206	77.2	61	22.8	12.07 ± 3.11
Rules, standard operating procedures (PL)	4 – 20	195	73.0	72	27.0	12.19 ± 2.88
Laws, professional codes (PC)	4 – 20	213	79.8	54	20.2	8.79 ± 2.45
Organization profit (EL)	4 – 20	201	75.3	66	24.7	21.09 ± 4.23
**Overall Ethical Climate dimensions**	36 – 180	**207**	**77.5**	**60**	**22.5**	109.48 ± 18.73

***SD* Standard deviation**

Table 5 shows that, there were statistically significant positive correlations between efficiency dimension of ethical climate perception, overall innovative work behavior, and economic affairs and innovation dimension (*P* < 0.05). Also, there were statistically significant positive correlations between overall ethical climate perception and economic affairs and innovation dimension of innovative work behavior. Furthermore, there were statistically significant positive correlations between economic affairs & innovation, friendship, team interest dimensions of innovative work behavior, and between social responsibilities, personnel policy dimensions of ethical climate perception.

**Table 5:** Correlation between innovative work behavior dimensions and ethical climate dimensions perceptions (*n* = 267)

**Ethical climate dimension**		**Innovative work behavior dimension**							
		**Innovative work behavior daily activities**	**Employee production**	**Innovative output**	**Expected positive performance outcomes**	**Innovation stimulating leadership behavior**	**Personnel policy**	**Economic affairs & innovation**	**Overall**
**Self-interest (EI)**	**R**	-0.014	0.028	0.001	0.102	0.067	0.038	0.105	0.055
	**p**	0.822	0.654	0.981	0.097	0.274	0.541	0.086	0.370
**Efficiency (EC)**	**r**	0.011	0.054	0.029	0.058	0.153^*^	0.128^*^	0.166^*^	0.123^*^
	**p**	0.859	0.379	0.633	0.349	0.012^*^	0.037^*^	0.006^*^	0.044^*^
**Friendship, team interest (BI)**	**r**	-0.018	0.056	0.002	0.045	0.114	0.103	0.131^*^	0.090
	**p**	0.767	0.358	0.970	0.466	0.063	0.093	0.033^*^	0.141
**Social responsibility (BC)**	**r**	-0.049	0.007	0.023	0.015	0.094	0.131^*^	0.117	0.074
	**p**	0.424	0.907	0.708	0.801	0.126	0.032^*^	0.056	0.228
**Personal morality (PI)**	**r**	0.055	0.061	0.009	0.108	0.038	0.029	0.098	0.060
	**p**	0.368	0.317	0.881	0.079	0.532	0.641	0.110	0.333
**Rules, standard operating procedures (PL)**	**r**	0.017	0.045	-0.017	0.103	-0.010	0.001	0.062	0.018
	**p**	0.782	0.461	0.781	0.092	0.865	0.989	0.315	0.772
**Laws, professional codes (PC)**	**r**	-0.029	-0.005	-0.027	0.092	0.050	0.008	0.073	0.024
	**p**	0.643	0.935	0.657	0.135	0.416	0.894	0.231	0.699
**Organization profit (EL)**	**r**	0.057	0.075	0.031	0.072	0.062	0.079	0.104	0.084
	**p**	0.357	0.224	0.619	0.241	0.311	0.200	0.088	0.172
**Overall**	**r**	0.009	0.060	0.011	0.098	0.091	0.086	0.130^*^	**0.087**
	**p**	0.884	0.330	0.858	0.111	0.138	0.163	0.033^*^	**0.155**

r: Pearson coefficient

^*^Statistically significant at *p* ≤ 0.05

In addition, there were statistically positive correlations between efficiency dimension of ethical climate perception and innovation stimulating behavior, personnel policy, economic affairs & innovation dimensions sequentially (*P* < 0.05).

Finally, there wasn’t statistically correlation between innovative work behavior and ethical climate perception.

## Discussion

The present study was conducted to explore the relation between innovative work behavior and ethical climate perceptions among nursing personnel. Nurses, innovations in the healthcare industry are crucial for addressing some of the issues within the current system such as market competitiveness, difficulties in workplace and in education. All new candidates of nursing profession could have a great interest in innovation when the opportunities for innovation arise [26]. A high ethical climate perception allows nursing staff to adjust their behavior to follow moral guidelines for the growth of themselves, their colleagues, and their institution [19].

According to the foregoing study results, the highest percent of the studied sample indicated that they had positive perception about expected positive performance outcomes. Meanwhile, more than half of them indicated that their perception about personnel policy was negative. This may be due to the belief that innovation is usually related to positive performance outcome. Meanwhile, policies take long years to be renewed and nurses are not allowed to participate in setting them. This result disagreed with [27] who declared in their study that organization policies were the highest level of perception among all factors which affect the nurses, performance.

Regarding the distribution of innovative work behavior dimensions, the Innovative output dimension recorded the highest mean score. Meanwhile, the lowest mean score was recorded to expected positive performance outcome dimension. This is due to the nurses had a lot of new ideas; but they not find enough support from their supervisors for their innovations. This result was matched with a research conducted in Egypt by [28] who reported that innovation output recorded the highest agreement of staff nurses about innovative behavior domains. Also, with [29] who conducted their study in Saudi Arabia and found that Idea search domain had the highest score.

Concerning the perception of innovative work behavior, the present study indicates that there is more than half of studied sample has a positive perception of innovative work behavior. This may be to the availability of opportunities for staff nurses for innovations. Also, the younger a person is, the more the inclination for innovations. This result is matched with [9] who reported that both nurses’ and doctors’ assessments of innovative work behaviors are positively correlated with nurses’ knowledge, skills, abilities, values, and personalities.

Regarding ethical climate perception, the study results revealed that more than three quarters of nursing personnel had a negative perception of ethical climate. This may be due to the presence of some factors such as the shortage of nursing personnel, heavy workload, hospital system, and lack of experience of nurses that lead them to face ethical problems in their clinical practice. In addition, the difficulty of economic conditions and the nurses’ preoccupation with conflicts between each other’s that may not provide them the opportunity to make the ethical climate. This finding was consistent with a national study conducted in Egypt by [30] cleared that the highest percent of their nursing staff participants had a negative perception about ethical climate.

Concerning the distribution of the ethical climate dimensions, the study results indicated that most of the studied sample has a negative perception about self-interest dimension; this may be due to heavy the shortage of staff nurses and heavy workload, this result agreed with [24] who confirmed that the most positive perception of participants was to patients.

In addition, the present study found that nearly the third of studied sample revealed that their perception of efficiency was positive, which result in a dissatisfaction with their practice may because they get the work done quickly that make the environmental work pressure of them. Similarly, with [31] who revealed that staff nurses were dissatisfied with their own practice, and they stated that their performance efficiency needs to improve.

Regarding the dimensions of ethical climate perception, the present study indicates that the lowest score was related to laws, professional codes dimension; this may be due to the mangers, focus on performance and the heavy workload which prevent nurses from assuring their interest to laws and professional codes. On the contrast, these results not matched with [19] who confirmed that law and professional codes dimension was the highest average. In the same line, these results were inconsistent with [32] who studied nurses, perceptions about ethical climate in Midwives in obstetrics and pediatrics hospital in Turkey and showed that the independence climate was the lowest score.

Contrarily, this result was in disagreement with [33] who reported that the nurses, perception in general about ethical climate was moderately high. Also, this result was inconsistent with [34] who illustrated that the ethical climate was assessed by their participants as moderate positive.

Finally, the present study results revealed that there was a positive statistical correlation between efficiency sub dimension and the overall Innovative work behavior. Also, there was a positive statistical correlation between the Economic Affairs and Innovation sub dimension and the overall ethical climate perception, this may be due to the more the efficiency of the employee, the greater the ability for innovations at work and when there is an abundance of resources, this will lead to a greater commitment of professional ethics by nurses.

These results were consistent with [19] who indicated that there was a positive and significant correlation between the health workers, levels of innovative work behavior and independence that can lead to efficiency. In the same line [28], reported that providing nurses with conductive work environment where they can reach their fullest potential is very important to enhance their innovation. Similarly, these results agreed with [35] in their study and found that there was a moderate and positive correlation between the behavior of managers, leaders and the work motivation of nurses.

Additionally, there were statistically significant correlations between Economic Affairs, innovation, friendship and team interest sub dimensions, and between social responsibility and personnel policy sub dimensions. This may be due to the availability of economic resources in the workplace can lead to a climate that is free from conflicts and encourages friendship and teamwork. Also, when nurses become more knowledgeable about the organizational policies, it will improve their social responsibility.

In the same line [36] who indicated that every nurse must become aware of the resources available in their practice environment for handling any ethical dilemmas, learn how to use them, and work with their coworkers. Similarly, these findings were consistent with [37] who cleared that all domains of the organizational culture (which build group cohesiveness) was significantly correlated with individual innovation.

Also, there were statistical correlations between efficiency sub dimension and innovation stimulating leadership behavior, personnel policy and economic affairs and innovation sub dimensions sequentially. This may be due to the higher the leader’s stimulation for his employees, the higher their efficiencies. Also, the presence of abundance of resources and good policies lead to greater satisfaction by nurses that positively affect their efficiencies. This result was in agreement with [38] they showed in their research that when nurse supervisor used the transformational leadership style, nurses became more independent and had high satisfaction during performing their duties.

### Limitations

This research has several limitations. First, there may be bias in the participants, responses due to the use of self-reported questionnaires. Second, Using limited size of the study sample; this may limit the results from generalization. Third, the translation process of the questionnaires did not follow a rigorous process and may need to be confirmed in future research.

## Conclusion

Based on the results obtained by the current study results, it can be concluded that more than half of nursing personnel had a positive perception of innovative work behavior and more than three quarters of them had a negative perception of ethical climate. Moreover, there were statistically significant positive correlations between efficiency dimension of ethical climate and the innovation stimulating leadership behavior, personnel policy, economic affairs & innovation, overall innovative work behavior perception sequentially. Additionally, there was a statistically significant positive correlation between the economic affairs & innovation dimension and the overall ethical climate perception (*P* < 0.05).

## Recommendations

The following recommendations are based on the findings of the current study where the nursing managers should be provide their nursing personnel with all opportunities and resources for initiating innovative behaviors, provide their nursing personnel with opportunities to participate and discuss Personnel policies which affect them. Maintain adequate nursing personnel for all units. Encourage nursing personnel for socialization and teamwork. Try to solve any conflict or ethical dilemma facing nurses in a constructive manner. Provide nursing personnel with ethical standards and regulations book. Encourage nursing personnel to discuss their self-interests and create an ethical and creative atmosphere at work by involving them in making decisions that concern them and providing the opportunity for training and improvement.

## Future research

From our research we can expect the future research to be the following: Examine factors influencing nursing ethical climate. Explore the relation between leadership style and ethical climate perception. Explore strategies that enhance innovation among nursing staff, and explore the intern students, perceptions about ethical climate.

## Data Availability

The data analyzed during the current study is available from the corresponding author on reasonable request.

## Abbreviations

IWB: Innovative Work Behavior
ICU: Intensive Care Unit
OR: Operating Room
NICU: Neonate Intensive Care Unit
PICU: Pediatric Intensive Care Unit
IWBQ: Innovative Work Behavior Questionnaire
